# Deep Learning Algorithm to Determine the Presence of Rectal Cancer from Transrectal Ultrasound Images

**DOI:** 10.3390/life15091358

**Published:** 2025-08-27

**Authors:** Min Cheol Chang, Sung Il Kang, Sohyun Kim

**Affiliations:** 1 Department of Rehabilitation Medicine, College of Medicine, Yeungnam University, Daegu 42415, Republic of Korea; wheel633@gmail.com; 2 Department of surgery, College of Medicine, Yeungnam University, Daegu 42415, Republic of Korea; sungiry@naver.com

**Keywords:** rectal neoplasm, transrectal ultrasound, deep learning, neural network, diagnosis

## Abstract

Background: Transrectal ultrasound (TRUS) is a crucial diagnostic tool for accurately detecting rectal cancer; however, its accuracy varies with the examiner’s experience. Deep learning, particularly convolutional neural networks (CNNs), exhibited promise in improving diagnostic accuracy in medical imaging. This study developed and assessed a CNN model for identifying rectal cancer using TRUS images. Methods: We retrospectively gathered 681 TRUS images that were obtained between August 2008 and September 2022. The images were classified as rectal cancer and normal rectum. Then, a CNN model was trained using the EfficientNetV2-S architecture to differentiate between rectal cancer and normal rectum images. Results: Of the 681 TRUS images, 533 and 148 were obtained from rectal cancer and normal rectum cases, respectively. The CNN model achieved training and validation accuracies of 96.7% and 90.5% and areas under the curve of 0.996 and 0.945, respectively. The precision, recall, and F1 scores were 0.935, 0.944, and 0.940 for rectal cancer and 0.793, 0.767, and 0.780 for the normal rectum, respectively. Conclusions: Our CNN model exhibited good performance in distinguishing rectal cancer from the normal rectum in TRUS images. The model is a valuable decision-support tool to help clinicians. Future studies are warranted to improve the model’s generalizability and enable stage classification integration.

## 1. Introduction

Rectal cancer, a rectal malignancy, is a significant global health concern because of its high incidence and related mortality rates [1]. Globally, it is the third most prevalent cancer and one of the leading causes of cancer-related deaths [2]. In some countries, such as Hungary and Korea, colorectal cancer rates were age-standardized at 51.2 and 44.5 per 100,000 people, respectively [3]. In 2020, 71.6% of all colorectal cancer cases were diagnosed at the age of 50–75 years [4]. However, incidence was increasing in younger populations, which is expected to contribute to the global burden [4]. Early detection and accurate diagnosis are crucial in improving patient outcomes, as timely intervention significantly reduces morbidity and mortality [4]. Rectal cancer often presents with subtle symptoms in its early stages, making early diagnosis without the use of advanced imaging techniques challenging [5]. The predominant clinical manifestations of colorectal cancer include abdominal pain (55%) and rectal bleeding (46%) [6]. Patients delay seeking medical attention for up to 6 months due to non-specific symptoms [7].

Traditionally, rectal cancer diagnosis relies on a combination of clinical assessment, biopsy, and imaging methods [8]. However, clinical staging for rectal cancer is identified exclusively by radiologic assessment. Patients usually undergo magnetic resonance imaging (MRI) or TRUS to identify the preoperative treatment according to clinical staging [9]. Both MRI and ultrasonography (US) exhibited a high accuracy for clinical staging. MRI accuracy was reported at 65% for T3 tumors and 80% for T4 tumors [10]. MRI accuracy for lymph node (LN) was 74.1% of node-negative tumors and 44.4% of node-positive tumors [10]. TRUS demonstrated approximately 80% of T3 tumors [11]. TRUS provided better information than MRI for tumor size and location [12]. TRUS is particularly effective in identifying the depth of invasion and the presence of LN metastasis, which are crucial factors in staging the disease and planning treatment [13,14]. Both MRI and US are available tests, but with limitations. MRI is relatively expensive and time-consuming, and it is contraindicated in some patients. TRUS has appeared as a valuable non-invasive tool for evaluating rectal lesions and assessing tumor characteristics [13,14]. Moreover, TRUS is relatively inexpensive, non-invasive, and performed quickly in an outpatient clinic. However, the diagnostic accuracy of TRUS varies based on the examiner, and improving the diagnostic accuracy of TRUS requires a lot of training and effort. We have considered applying machine learning (ML) to strengthen the benefits and restrict the limitations of TRUS. Few studies have applied ML-based TRUS in rectal cancer. However, artificial intelligence-assisted endoscopic US has been reported to improve the diagnostic accuracy of gastrointestinal stromal tumors [15]. Similarly, ML-enhanced TRUS may enhance the diagnostic accuracy of rectal cancer. With increased objectivity through ML, the use of TRUS may be extended to a wider range of patients.

ML is a computer algorithm that automatically learns from data without requiring explicit programming [16,17,18]. ML is popular for its ability to overcome the limitations of existing image analysis techniques and enable breakthroughs in the field of image analysis [16,17,18]. Deep learning (DL) is an advanced ML approach involving the use of a large number of hidden layers to establish artificial neural networks with structures and functions similar to those of the human brain [19,20,21]. It learns from unstructured and perceptual image data. Several studies have revealed that the DL technique outperforms traditional ML techniques [19,20,21]. CNN is a representative DL model that is specialized in image analysis [22,23]. Thus, a CNN model recognizes and analyzes the TRUS images and determines the presence of rectal cancer. Therefore, a CNN model could help physicians lacking experience with TRUS in the diagnosis of rectal cancer, potentially reducing misdiagnosis by physicians.

This study aimed to develop a CNN model to improve the accuracy of rectal cancer detection using TRUS images. We used TRUS images as input data to train a CNN model to identify the presence of rectal cancer.

## 2. Materials and Methods

### 2.1. TRUS Image Collection

**Figure 1:**
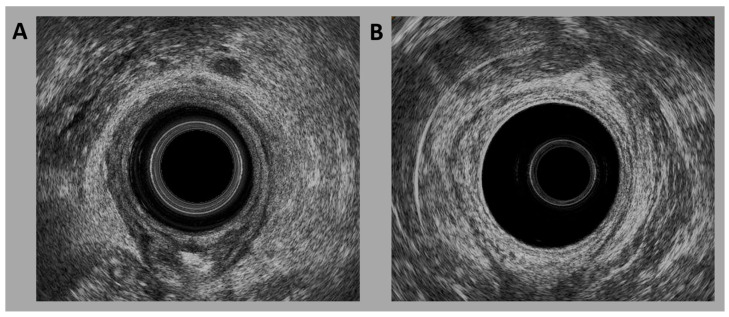
Transrectal ultrasound images: (**A**) rectal cancer and (**B**) normal rectum.

This study retrospectively recruited 681 patients who underwent TRUS in a single university hospital from August 2008 to September 2022 (Figure 1). A total of 712 consecutive patients who visited a university hospital underwent TRUS. Of these examinations, 681 rectal US images were retrospectively analyzed for this study. The inclusion criteria for rectal cancer imaging were (1) patients pathologically diagnosed with rectal cancer by endoscopy; (2) patients with rectal lesions located within 10 cm of the anal verge; and (3) patients with successful TRUS. Exclusion criteria for rectal cancer imaging were (1) patients with a suspected non-invasive mass on endoscopy and computed tomography and (2) those with unsuccessful TRUS. The inclusion criteria for normal rectum imaging were as follows: (1) patient absence of rectal abnormal lesions, (2) rectal lesions located within 10 cm of the anal verge, and (3) successful TRUS examination. The success of TRUS was defined as a complete assessment of the depth of invasion by passing the US probe through the entire mass. Each TRUS image was collected from a different assessment, and a single representative image was selected from each examination. The representative image for TRUS assessment was defined as demonstrating the maximal depth of tumor invasion. The obtained TRUS images (n = 681) were reviewed and categorized into rectal cancer and normal rectum images by colorectal surgeons with >10 years of experience using TRUS. The Yeungnam University Hospital’s IRB granted an exemption from written informed consent for protocol (2024-11-025), citing the study’s retrospective design. All images used in this study were de-identified before analysis, and no personal health information was retained. This study was conducted in accordance with the principles outlined in the Declaration of Helsinki.

### 2.2. TRUS

US was conducted using a three-dimensional (3D) US machine from Brüel & Kjae Medical (Naerum, Denmark), equipped with a 2052 endorectal probe (frequency 6–16 MHz). Although the US machine was replaced in 2017 with another unit from the same manufacturer, all examinations followed the same protocol. Patients received a cleansing enema before the assessment. The patients were placed in the left lateral position. Physicians performed a digital rectal examination before the US test. The probe was inserted through the anus, and 50 cc of water was injected into the probe once the patients were in the appropriate position. A 3D dynamic image scan was taken once the probe was positioned as deep as it could reach. Then 3D dynamic imaging was conducted, with the probe automatically rotating and scanning at 360°. The patient’s normal and lesioned areas were separately frozen, and each photo was saved. Rectal cancer typically manifests as a hypoechoic lesion that infiltrates and disruptes the normal layered architecture of the wall, frequently with an irregular and ill-defined outer margin (Figure 1A). Normal TRUS findings describe the absence of hypoechoic lesions involving the rectal wall and the preservation of distinct interfaces between hyperechoic and hypoechoic layers (Figure 1B).

### 2.3. DL Algorithms

Python version 3.10.12, SciKit-Learn version 1.5.2, and PyTorch version 2.4.1 with the timm library were utilized to develop the CNN model for identifying the presence of rectal cancer. The input data were 681 TRUS images, and the output data were categorized into a binary classification of rectal cancer and the normal rectum. We utilized the EfficientNetV2-S model to establish a model to determine the presence of rectal cancer in the TRUS images (Table 1). The EfficientNetV2-S architecture offers a favorable balance between model complexity and accuracy, with improved training speed and parameter efficiency compared to other well-established CNNs such as ResNet or DenseNet.

All 681 TRUS images in our study were split using an 80:20 holdout approach. Specifically, a stratified split based on labels was performed, generating 544 (79.88%) and 137 images (20.12%) for the training and the validation sets, respectively. No separate independent test set was conducted, and final model performance was assessed using the validation dataset.

All the TRUS images used for model training were resized to 224 × 224 pixels to match the input requirements of EfficientNetV2-S. Normalization was performed using the ImageNet dataset’s channel-wise mean and standard deviation (mean = 0.485, 0.456, and 0.406; standard deviation = 0.229, 0.224, and 0.225). We applied random horizontal flip, random rotation (±10°), and random resized crop techniques during training for data augmentation to improve generalization. A range of optimizers, learning rates, and batch sizes were employed, incorporating dropout regularization to prevent overfitting. Table 1 shows the details of the proposed model. Although the model architecture, training, and evaluation were implemented in PyTorch version 2.4.1, with the timm library, Keras Tuner was employed exclusively for hyperparameter optimization. A custom training loop in PyTorch was wrapped and called with the Keras Tunner search process, enabling us to utilize its efficient search algorithms while implementing a PyTorch-based model. This hybrid approach allowed flexible model development and systematic hyperparameter tuning.

### 2.4. Statistical Analysis

Statistical analyses were conducted using Python version 3.10.12 and Scikit-Learn version 1.5.2. Receiver operating characteristic (ROC) curve analysis was conducted, and the area under the curve (AUC) was calculated. The 95% confidence interval (CI) for the AUC was calculated as described by DeLong et al. [24]. Scikit-Learn was used to calculate the ROC curve and AUC. Further, we assessed the accuracy, precision, recall, and F1 score of our developed model. The accuracy is the proportion of correct predictions (both true positives and true negatives) among the total number of cases investigated. The precision is the ratio of true positive predictions to the total number of instances classified as positive by the model. The recall, also known as sensitivity or the true positive rate, measures the proportion of actual positive instances correctly determined by the model. The F1 score is the harmonic mean of precision and recall, providing a single metric that balances both concerns.

## 3. Results

Of the 681 TRUS images, 533 were images of rectal cancer (mean age = 62.50 ± 10.80 years, M:F = 374:159, T0:T1:T2:T3:T4 = 7:43:134:313:36), and 148 were images of the normal rectum (mean age = 61.06 ± 10.36 years, M:F = 94:54).

**Figure 2:**
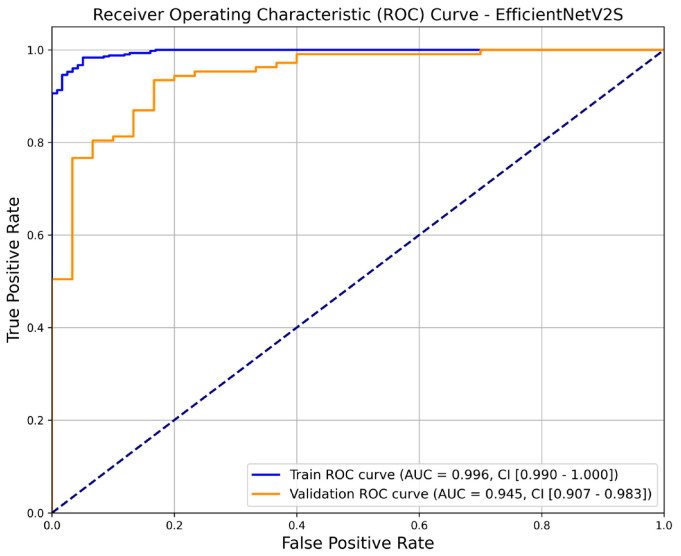
Receiver operating characteristic curves for the training and validation datasets of the proposed model. AUC, area under the curve; CI, confidence interval.

Table 2 summarizes the sample characteristics of the proposed model. A total of 681 TURS images were included in this analysis, comprising 544 (79.88%) in the training set and 137 (20.12%) in the validation set. In the training set, rectal cancer and normal rectum cases accounted for 78.31%and 21.69%, respectively, whereas in the validation set, the proportions were 78.10% and 21.90%, respectively. The trained model demonstrated robust performance with a training accuracy of 96.7% and AUC of 0.996 (95% CI: 0.990–1.000). Additionally, the validation accuracy was high at 90.5%, with an AUC of 0.945 (95% CI: 0.907–0.983) (Figure 2).

The model demonstrated good performance in classifying rectal cancer, with precision, recall, and F1 scores of 0.935, 0.944, and 0.940, respectively. The precision, recall, and F1 score for the normal rectal set were 0.793, 0.767, and 0.780, respectively. The macro-averaged precision, recall, and F1 score across both sets were 0.864, 0.855, and 0.860, respectively, indicating balanced performance at the set level.

**Figure 3:**
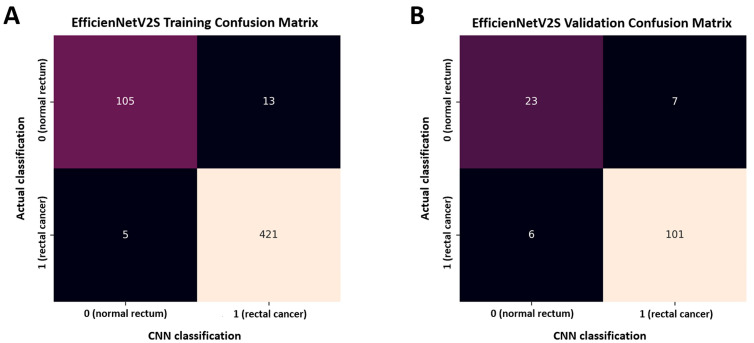
Confusion matrix for the proposed model: (**A**) training data and (**B**) validation data.

Figure 3 shows the model’s characteristics using a confusion matrix analysis of the validation data. The confusion matrix demonstrates that the model correctly identified 101/107 rectal cancer cases (94.4%). Additionally, the model correctly predicted 23/30 normal rectum cases (76.7%).

## 4. Discussion

This study developed a CNN model to identify the presence of rectal cancer from TRUS images and assessed its performance. Our model achieved a validation accuracy of 90.5% and an AUC of 0.945. The ability of our model to distinguish between rectal cancer and normal rectum on TRUS images is encouraging, considering that AUCs of 0.7–0.8, 0.8–0.9, and >0.9 are generally considered acceptable, excellent, and outstanding, respectively [25,26]. These results indicate that DL, particularly CNNs, can be used effectively to detect rectal cancer on TRUS images, potentially improving diagnostic accuracy and helping clinicians. Few studies have investigated the application of DL-based TRUS in rectal cancer. Our results indicate that this approach is feasible and holds promise for delivering more accurate clinical information to guide patient management.

The class-specific performance measures were encouraging. The model demonstrated good discrimination in rectal cancer detection, demonstrating favorable sensitivity and specificity. In contrast, its ability to accurately identify normal rectal tissue was relatively limited. This indicates that the model was relatively good at identifying rectal cancer, but it had more difficulty distinguishing normal rectum images. However, these findings remained clinically valuable, as achieving high sensitivity for cancer detection was crucial in clinical practice.

Few studies have investigated the use of DL to improve the accuracy of TRUS in rectal cancer. An ML model incorporating TRUS and contrast-enhanced US helped in the preoperative prediction of regional LN metastasis [27]. The majority of DL research in TRUS has focused on prostate cancer, precluding direct relevance to rectal cancer [28,29]. TRUS is employed in prostate cancer evaluation, but its primary function is lesion detection and biopsy guidance. In contrast, TRUS in rectal cancer serves to assess the extent of tumor infiltration through the rectal wall. Direct comparisons between the two applications are limited by their fundamentally different diagnostic purpose. However, studies in prostate cancer have demonstrated that ML-enhanced TRUS offers excellent predictive accuracy and intraoperative procedures, indicating that comparable benefits may be achievable in rectal cancer [29,30]. Although not specific to TRUS, the integration of DL with other US modalities has been explored across medical fields. In endoscopic US, AI assistance has been reported to improve diagnostic accuracy, particularly for gastrointestinal stromal tumors [15]. Additionally, studies have examined the performance of combining DL and US in the breast, thyroid, kidneys, etc. [31,32,33]. These advancements suggest that DL-enhanced TRUS could enhance the accuracy and objectivity of rectal cancer assessment.

To use our developed model in real-world clinical practice, we need to conduct a clinical study to validate the model’s diagnostic accuracy for rectal cancer. Further, we must make further efforts to improve its diagnostic accuracy. Once validated, we can deploy the model via an application programming interface to enable its application in clinical settings.

This study has several limitations. First, its comparatively lower performance in identifying normal rectal tissue and tumor staging emphasizes the need for larger, multicenter datasets to improve generalizability and staging accuracy. The analysis of a greater number of TRUS images from each cancer stage may achieve accurate stage classification. Second, selecting a single representative image from each TRUS examination introduces potential selection bias. Third, the dataset exhibited a class imbalance (533 rectal cancer images vs. 148 normal rectum images), which likely reduced classification performance of the normal rectum. This was further affected by the analysis of fewer normal rectum images, and no class-balancing techniques, such as class-weighted loss or oversampling, were employed. Fourth, the gap between training (AUC 0.996) and validation (AUC 0.945) performance indicates a potential risk of overfitting. This may have been amplified by the relatively small dataset size, the absence of an independent or external test set, and the fact that data were obtained from a single university hospital. Thus, the current results might overestimate the true generalizability of the model. Finally, we did not explore the capacity of various DL algorithms other than EfficientNetV2-S, and interpretability techniques such as Grad-CAM, Shapley Additive Explanation, or Local Interpretable Model-agnostic Explanations were not applied. This limited our ability to visualize and confirm the model’s decision-making process.

The development of DL-based TRUS in rectal cancer may help diagnose mid-to-lower rectal tumors without requiring costly imaging modalities such as computed tomography or MRI. As this technology continues to advance and its accuracy improves, it can provide reliable clinical staging, thereby enabling appropriate preoperative treatment for suitable patients. Given the high cost, limited availability, and MRI contraindication in certain patients such as those with coronary stent, pacemakers, etc., DL-based TRUS may serve as a practical alternative in selected clinical conditions. The ability to automate the detection and classification of rectal cancer in TRUS images could not only improve the diagnosis speed but also help overcome some of the limitations associated with the human interpretation of US images. This is particularly beneficial in settings with limited access to expert radiologists or colorectal surgeons. Furthermore, the DL model helps reduce the likelihood of misdiagnosis, thereby providing a second layer of analysis to support a clinician’s judgment.

## 5. Conclusions

Our CNN model achieved relatively good performance in detecting rectal cancer on TRUS images, indicating potential as a decision-support tool for clinicians. However, its performance was evaluated only on a dataset from a single center, which limits generalizability and may overestimate real-world utility. Additionally, the model currently detects only the presence of tumors without staging capability. Future studies using larger, multicenter datasets and external validation are needed to confirm clinical applicability and to explore multiclass classification, including tumor staging.

## Figures and Tables

**Table 1:** Architecture of the proposed model.

Layer (Type)	Output Shape	Parameters
StochasticDepth	7 × 7 × 256	0
MBConv	7 × 7 × 256	0
Conv2d	7 × 7 × 1280	327,680
BatchNorm2d	7 × 7 × 1280	2560
SiLU	7 × 7 × 1280	0
AdaptiveAvgPool2d	1 × 1 × 1280	0
Linear	2	2562
Total parameters		20,180,050
Trainable parameters		20,180,050
Non-trainable parameters		0

**Table 2:** Performance of the proposed model on the validation dataset.

Sample size (patients)	544, 79.88% for training, 137, 20.12% for validation, total 681				
Sample ratio (patients)	Rectal cancer: 533, 78.27%; normal rectum: 148, 21.73%;				
	Rectal cancer: 426, 78.31%; normal rectum: 118, 21.69% for training				
	Rectal cancer: 107, 78.10%; normal rectum: 30, 21.90% for validation				
Model details	EfficientNetV2-S CNN model with full learning (unfreeze all layers)				
	Adam optimizer, SiLU activation				
	Learning rate 1 × 10^−3^, batch size 32, Epoch 25				
	Batch normalization and dropout for regularization				
	Image resized to 224 (H) × 224 (W)				
	Training accuracy: 96.7%, AUC 0.996 with 95% CI [0.990–1.000]				
	Validation accuracy: 90.5%, AUC 0.945 with 95% CI [0.907–0.983]				
	Class	Precision	Recall	F1-score	Support
Model performance (validation data)	Rectal cancer (1)	0.935	0.944	0.940	107
	Normal rectum (0)	0.793	0.767	0.780	30
	Macro average	0.864	0.855	0.860	137

AUC, area under the curve; CI, confidence interval; CNN, convolutional neural network; SiLU, sigmoid linear unit.

## Data Availability

Data is unavailable due to privacy or ethical restrictions.

## Abbreviations

The following abbreviations are used in this manuscript:

TRUS	Transrectal ultrasound
CNNs	convolutional neural networks
T	Tumor
LN	Lymph node
MRI	Magnetic resonance imaging
US	Ultrasonography
ML	Machine learning
DL	Deep learning
ROC	Receiver operating characteristic
AUC	Area under the curve
